# Temporary or Plastic Fillings

**Published:** 1877-09

**Authors:** Milton H. Chappell

**Affiliations:** Knightstown, Ind.


### TEMPORARY OR PLASTIC FILLINGS.

BY MILTON H. CHAPPELL, KNIGHTSTOWN, IND.

The merits of the different materials for plastic filling as offered by vendors to the profession, are of secondary consideration with our present subject. While it is true, the indications for the use of gutta percha, is different from the use of cements, such as "os-artificial," "Cement Plombe," or any of the preparations of oxi-chloride of zinc.

With observations in eighteen years' practice, I am convinced that a great number of failures in filling teeth, can be attributed to the indiscriminate use of gold, or amalgams, disregarding the pathological conditions of the teeth, and oral secretions, whether constitutional or local—the decay *black*, brown, *yellow-tinge*, or white.

It is a safe rule to adopt. *Fill no tooth with gold or amalgam, and consider it safe, unless the decay be black, or dark brown, dentine dense in structure, non-sensitive, the enamel of flint-like hardness. Teeth free from tartar and self cleansing, with the oral secretions normal.*

Every operator must admit that the teeth of *white decay*, are those with which failures mostly occur.

The excellence of the operation being the same in either case.

A multiplicity of causes may exist, but in a majority of cases, if not the entire number operated upon, we can control, and after proper treatment class them with teeth that have had black decay and permanent operations.

We find it necessary to inquire into the general health of our patients, the oral anatomy and secretions, to ascertain the cause of the *white* or *yellow tinge* to *light brown decay* of the teeth.

After we have thus become acquainted with the pathology of our case, we then devise the remedies to correct the malady, whether for immediate or predisposing cause, or both, and the indications for temporary or plastic fillings.

The predisposing or constitutional tendencies may result from protracted illness, false diatetic life, new assimilation of bone phosphates in the pabulum for tooth structure, during the period of gestation and lactation.

The treatment must be in accordance with the demands or capabilities of the vital functions. Plain nourishing food, using graham, rye, or corn-bread, fresh milk and butter, when using meats or vegetables be as sparing of *salt* as the palate will permit, deserts of graham mush, with cream and sugar, observing regular meals with little variety at each meal. Eat sparingly, and no mincing between meals, take exercise in open air, but not to exhaustion. Bathe frequently and regularly, and the predisposing tendencies will rapidly change for the better.

At our first appointment we must operate for the immediate cause—uncleanliness, resulting from irregular teeth by particles of food, mucus and etc., lodging between the teeth. Green stain, deposits of tartar, *Leptothrix Buccalis*, forming a nucleus for vitiation, and nascent acids, either nitric, or hydrochloric, forming white, yellow tinge, to light brown decay.

In *all cases if possible remove the immediate cause at first appointment* as before stated,—calculi, and affected margins of alveolar process, and polishing away all green stain. Cut away frail enamel with the chisel, file and sand paper, then remove all decayed material, and shape cavity for filling.

In dressing the cavity protect the tooth from moisture with the rubber dam securely adjusted. If you should find the tooth "chalky," caries deeply seated with *fresh white decay*, near approach to dental pulp, extreme sensitiveness, health impaired and with predisposing tendencies, you may rest assured that the carious affection penetrates the dentine, and on the highway to the pulp cells, (*odontoblasts*) if not already getting its "sharp shooters" to work, or the "Siege guns" bombarding the citadel —the pulp.

When we find the pulp protected by its wall of dentine, and the functions of the pulp cells in a normal condition the cavity comparatively small, wipe out with alcohol, dry with oxide of zinc then fill with oxy-chloride of zinc cement, either os-artificial, cement plombe or any other of the chloride combinations,—burnish well with hot instrument, and if the material sticks to the instrument, use soap-stone or any other dry lubricator, and you can make a very hard surface which will last until the predisposing tendencies change for the better;. which we find in the majority of patients from four to six months. Fillings so made may last for years.

In cases where caries are deep seated, near approach to pulp, thermal changes affecting the parts, *the cements cannot be tolerated*, owing to the irritating effects of the chloride, unless the communication is absolutely cut off, the use of gutta percha therefore is indicated. and will give the best results. In many of these cases where the patient has experienced pain when foreign substance is forced into the cavity, and on removing the offending substance the pain subsides. In this we have the irritating chemical agencies rapidly working destruction of the vascular tissues, known as sensitive or inflamed dentine, or of the pulp. If we are sure the trouble is only acute, septic treatment alone is required, after drying, fill with gutta percha. If we are treating the pulp, where both immediate and constitutional tendencies exist in the production of white decay, the recuperative power of the pulp is very meager in repelling or withstanding the approach of caries, or remaining normal under severe medication—*the tissues desire to be let alone*, and possibly *sedative* rest is necessary. Therefore in using non-corrosive material we enhance the probabilities of success with these hazardous cases.

By closely making a register of the class of decay, and its treatment, with subsequent results, I have formed these rules in practice, and can safely say that the number of teeth securely and permanently saved have been greatly increased, and might add that the teeth on examination after months of this temporary treatment present cavity walls free from caries. More dense in structure than when first operated upon, and if perchance not to my satisfaction, I repeat the temporary treatment until better results are obtained, *then fill permanently with gold*, (or with amalgam if tooth or patient will not admit of gold.)

Teeth of white decay are hazardous and fearfully uncertain of success if filled with metal at first appointment and the patient dismissed with the idea that the operation is complete and permanent, while the pathological and common sense facts are disregarded. We frequently have *fresh white decay* in the same mouth or tooth with *black decay*, in such cases the precautions of the white decay must be observed in the cavities of the white decay.

The status of our professional operations, are in accordance with our application of judgment founded on scientific principles and not by any sterreotyped professional ruts that so many have labored to overcome.

